# Editorial: Protein posttranslational modifications in plant responses to abiotic stress - Women in plant science series

**DOI:** 10.3389/fpls.2022.1049173

**Published:** 2022-10-11

**Authors:** Agnieszka Sirko, Cecilia Gotor, Anna Wawrzyńska

**Affiliations:** ^1^ Laboratory of Plant Protein Homeostasis, Institute of Biochemistry and Biophysics Polish Academy of Sciences, Warsaw, Poland; ^2^ Instituto de Bioquímica Vegetal y Fotosíntesis, Consejo Superior de Investigaciones Científicas (CSIC) and Universidad de Sevilla, Sevilla, Spain

**Keywords:** protein posttranslational modifications (PTMs), abiotic stress, S-Nitrosylation and nitration, ubiquitylation (Ubiquitination), cysteine oxidation, S-Acylation, selenium toxicity

Abiotic stressors such as low nutrient availability, drought, flood, salinity, extreme temperatures, or high UV regularly affect plant growth and development causing severe agricultural production limitations around the world. Post-translational modifications (PTMs) are often involved in plants’ response to stresses enabling the coordination of multiple signaling pathways. The mechanisms and dynamics of PTMs are now a crucial topic of plant research because they represent one of the quickest and earliest responses to environmental changes. PTMs expand proteome diversity and enlarge its functionality affecting the signaling pathways, gene expression, protein stability, localization and interactions, and enzyme kinetics, all at relatively low costs for the cell. Therefore, to obtain a response that is distinct to the type of stress and its duration, plant signaling pathways mainly rely on modifications of particular amino acid residues in proteins. Although the list of PTMs in the plant proteome is growing rapidly, it is often unclear how they affect proteins and their function.

The goal of this Research Topic is to provide an overview of the recent work of experts studying PTM’s role in plants, with a special emphasis on the processes involved in abiotic stress signaling and response.

Among many mechanisms of reactive oxygen and nitrogen species sensing in cells, their reactivity with cysteine residues appears to be a major oxidative PTM among living kingdoms (Mukherjee, 2020). The formation of a wide range of cysteine PTMs, including S-nitrosylation (-SNO), S-glutathionylation (-SSG), persulfidation or S-sulhydration (-SSH), sulfenylation (-SOH), sulfinylation (-SO2H), and sulfonylation (-SO3H), is possible due to the various oxidation states of sulfur. Selles et al. describe a crucial role of cysteine in Arabidopsis parvulin PIN1At, a protein with prolyl isomerase activity, which catalyzes the *cis-trans* isomerization of proline peptide bonds. The authors prove that a single cysteine residue (Cys69) in PIN1At is sensitive to peroxide-dependent oxidation leading to the formation of covalent dimers, or at higher excess of H_2_O_2_ to sulfinic, or sulfonic forms that inhibit protein activity *in vitro*. In many eukaryotic cells, selenium is a crucial trace element required for the production of seleno-proteins, however, its excess is often toxic. This toxicity is at least partly caused by the misincorporation of Se-Cys into proteins (Schiavon and Pilon-Smits, 2017), which due to the different redox properties affects disulfide-bridge formation essential for protein tertiary and quaternary structure formation. Khan et al. found that Arabidopsis plants can discriminate between selenite and selenate which is predominantly stored in leaves causing chlorosis. Surprisingly, they discovered that selenite oxidized the plastidic glutathione pool located in roots while the cytosolic glutathione pool was predominantly oxidized by selenate, though the plastidic redox environment in leaves was similarly compromised. Prior to this study, the selenate-induced impairment of the glutathione redox milieu remains unidentified, in contrast to the known effect of selenite.

A series of reviews in this Research Topic also add to the current knowledge of PTMs function in the regulation of plant development and stress responses. The three reviews stay on the subject of cysteine modification. Li et al. sum up the latest findings on protein S-acylation. The addition of 16-carbon palmitate or 18-carbon stearate is reversible and offers a molecular mechanism for the cycling and trafficking of membrane-associated proteins between various cell compartments. The authors nicely describe protein S-acyltransferase (PAT) family proteins as well as de-S-acylation enzymes in Arabidopsis and other higher plants. However, precise regulatory mechanisms for PAT and de-S-acylation enzymes activities, substrate specificity, and responses to external stimuli are still poorly understood. In another review, Bont et al. summarized the known oxidative modifications affecting cysteine residues of the enzymes involved in sulfate assimilation and the synthesis of cysteine, methionine, and glutathione in plants. Such regulatory redox switches are essential for sulfur division between the primary and secondary pathways of sulfur assimilation, especially when the demand for reduced sulfur to support cysteine and glutathione synthesis increases, for example under oxidative stress conditions. The authors underly a prominent regulatory role of cysteine persulfidation which might arise from the fact that sulfide is produced by the primary sulfate assimilation pathway therefore it may react with oxidized thiol groups of the pathway enzymes. Nitric oxide as a free radical is very reactive and targets also cysteine leading to its S-nitrosylation. Pande et al. describes the effect of such modification on phytohormone signaling. Depending on the response necessary to maintain cellular homeostasis, essential proteins engaged in the phytohormonal network may be S-nitrosylated during their synthesis, degradation, or signaling activities. Step by step, the authors present S-nitrosylated proteins involved in the auxin, gibberellic acid, salicylic acid, jasmonic acid, abscisic acid, cytokinin, ethylene, brassinosteroid, and strigolactone signaling, showing the dual role of nitric oxide in the up and downregulation of plant hormones. The subject of the next review by Leon focuses on another modification caused by nitric oxide - the nitration of tyrosine and the impact of such PTM on nitric oxide signaling. A nice summary of identified nitrated plant proteins and the functional effects on the modified proteins is presented. Additionally, Leon resumes the current methodologies to study protein nitration underlying the importance of the genetic code expansion technologies.

Finally, the last review focuses on the importance of ubiquitination and subsequent degradation through 26S proteasome in facilitating changes to the proteome necessary to lessen the negative effects of abiotic stressors on plants. Protein ubiquitination consists of the reversible, covalent binding of a single or a chain of ubiquitin molecules to lysine residues of the protein (Vierstra, 2012). The fact that approximately 7% of all Arabidopsis coding genes are predicted to be involved in protein ubiquitination highlights its enormous impact on plant growth and development (Friso and van Wijk, 2015). Mackinnon and Stone discuss the role of just several E3 ubiquitin ligases involved in regulating nutrient uptake and responses to selected abiotic stresses like nutrient deficiencies.

We are appreciative of each author’s contribution to this Research Topic. We also like to thank all of the reviewers for their time and effort in evaluating the work presented here and shaping this focus Research Topic.

## Author contributions

AW drafted the manuscript; AS and CG have made a substantial, direct, and intellectual contribution to the work and approved it for publication.

## Funding

AS and AW acknowledge funding from National Science Centre in Poland (grant nos: 2018/31/F/NZ1/02234 and 2021/41/B/NZ3/02788); CG acknowledges funding from ERDF A way of making Europe and MCIN/AEI/10.13039/ 501100011033 (grant No. PID2019-109785GB-I00), and Junta de Andalucía (grant No. P18-RT-3154).

## Conflict of interest

The authors declare that the research was conducted in the absence of any commercial or financial relationships that could be construed as a potential conflict of interest.

## Publisher’s note

All claims expressed in this article are solely those of the authors and do not necessarily represent those of their affiliated organizations, or those of the publisher, the editors and the reviewers. Any product that may be evaluated in this article, or claim that may be made by its manufacturer, is not guaranteed or endorsed by the publisher.
